# Metabolomic Signatures of Recovery: A Secondary Analysis of Public Longitudinal LC–MS Datasets Shows Polyphenol-Rich Interventions Attenuate Purine Degradation and Oxidative Stress Following Exhaustive Exercise

**DOI:** 10.3390/metabo16010079

**Published:** 2026-01-16

**Authors:** Xuyang Wang, Chang Liu, Yirui Chen, Mengyang Wang, Kai Zhao, Wei Jiang

**Affiliations:** 1 China Volleyball College, Beijing Sport University, Beijing 100084, China; wxy333221@163.com; 2 School of Sport Science, Beijing Sport University, Beijing 100084, China; c.liu@bsu.edu.cn; 3 College of Public Health and Health Sciences, Tianjin University of Traditional Chinese Medicine, Tianjin 301617, China; 13385883334@163.com (Y.C.);

**Keywords:** nutraceuticals, recovery kinetics, xanthine oxidase pathway, redox homeostasis, lipid peroxidation, systems metabolomics, network analysis

## Abstract

**Background:** Post-exercise recovery involves coordinated metabolic restoration and redox rebalancing. Although dietary polyphenols have been proposed to facilitate recovery, the metabolic mechanisms underlying their effects—particularly during the recovery phase—remain insufficiently characterized. This study aimed to investigate how polyphenol supplementation modulates post-exercise metabolic recovery using an integrative metabolomics approach. **Methods:** We conducted a secondary analysis of publicly available longitudinal human LC–MS metabolomics datasets from exercise intervention studies with polyphenol supplementation. Datasets were obtained from the NIH Metabolomics Workbench and MetaboLights repositories; study-level metadata were used as provided by the original investigators. Global metabolic trajectories were assessed using principal component analysis (PCA) and orthogonal partial least squares discriminant analysis (OPLS-DA). Targeted analyses focused on purine degradation intermediates and redox-related metabolites. Correlation-based network and pathway enrichment analyses were applied to characterize recovery-phase metabolic reorganization. **Results:** Exercise induced a pronounced global metabolic perturbation in both placebo and polyphenol groups. During recovery, polyphenol supplementation was associated with a partial reversion of the metabolome toward the pre-exercise state, whereas placebo samples remained metabolically displaced. Discriminant metabolite analyses identified purine degradation intermediates and oxidative stress-related lipid species as key contributors to group separation during recovery. Polyphenol supplementation attenuated recovery-phase accumulation of hypoxanthine, xanthine, and uric acid and was associated with a sustained suppression of the uric acid-to-hypoxanthine ratio. Network analyses revealed weakened correlations between purine metabolites and oxidative stress markers, along with reduced network centrality of stress-responsive metabolic hubs. **Conclusions:** These findings indicate that polyphenol supplementation is associated with accelerated metabolic normalization during post-exercise recovery, potentially through modulation of purine-associated oxidative pathways and system-level metabolic network reorganization.

## 1. Introduction

Strenuous exercise induces marked, transient shifts in systemic metabolism driven by acute energetic demand, localized hypoxia, and redox imbalance. While these perturbations are essential for physiological adaptation, incomplete or delayed metabolic recovery can exacerbate oxidative stress, impair tissue repair, and compromise subsequent performance [1]. Consequently, understanding the molecular processes governing post-exercise recovery has emerged as a critical challenge in exercise physiology and metabolic health research.

Among nutritional strategies proposed to facilitate recovery, dietary polyphenols have attracted considerable attention. Polyphenol-rich interventions have been reported to attenuate exercise-induced oxidative stress, inflammation, and muscle damage, with some studies suggesting improvements in recovery kinetics. However, findings across trials remain heterogeneous, likely reflecting differences in polyphenol composition, dosing, timing, and study design [2]. More importantly, despite accumulating evidence for beneficial effects, the metabolic mechanisms through which polyphenols influence post-exercise recovery remain incompletely understood, particularly at the system-metabolism level.

Metabolomics provides a powerful framework for characterizing exercise-induced metabolic perturbations and their resolution over time. Previous metabolomics studies have primarily focused on acute exercise responses or single time-point comparisons, often emphasizing energy substrates, amino acids, or lipid metabolism. In contrast, the recovery phase—during which energetic restoration and redox rebalancing occur—has received comparatively less mechanistic attention, despite being a key determinant of adaptation and resilience [3,4]. Moreover, many prior analyses have relied on metabolite lists or pathway enrichment alone, offering limited insight into how metabolic processes interact and reorganize during recovery [5].

Purine metabolism represents a particularly compelling yet underexplored axis in this context. Exercise-induced nucleotide turnover leads to the accumulation of purine degradation intermediates, including hypoxanthine and xanthine, which are further oxidized to uric acid by xanthine oxidase (XO) [6]. This enzymatic process generates reactive oxygen species independent of mitochondrial respiration, thereby linking energetic stress to oxidative burden [7]. While purine metabolites have been consistently observed to increase following intense exercise, their role as potential drivers of sustained oxidative stress during recovery—and their modulation by nutritional interventions—has not been systematically investigated [8].

Beyond individual pathways, recovery from exercise is inherently a system-level process, involving coordinated interactions among energetic, redox, and biosynthetic networks [9]. Correlation-based network approaches offer a complementary perspective to traditional pathway analysis by capturing changes in metabolic connectivity and network topology [10]. Such approaches can identify highly connected metabolic hubs and reveal how interventions redistribute metabolic influence across pathways [11]. However, this system-level framework has rarely been applied to interrogate how polyphenol supplementation reshapes the metabolic architecture of post-exercise recovery.

In the present study, we performed an integrative secondary analysis of publicly available longitudinal human metabolomics datasets to investigate how polyphenol supplementation modulates post-exercise recovery [12]. By combining global multivariate modeling, targeted purine and redox profiling, and correlation-based network inference, we aimed to (i) characterize recovery-phase metabolic trajectories under polyphenol supplementation, (ii) evaluate the involvement of purine degradation and its association with oxidative stress, and (iii) determine whether polyphenols induce system-level metabolic network reorganization during recovery. Through this multi-layered analytical strategy, we sought to provide a mechanistic framework linking nutritional polyphenols to metabolic resilience following strenuous exercise.

## 2. Materials and Methods

### 2.1. Dataset Acquisition and Curation

We conducted a secondary analysis of publicly available longitudinal human LC–MS metabolomics datasets to evaluate how polyphenol supplementation modulates post-exercise metabolic recovery. Datasets were retrieved from the NIH Metabolomics Workbench and MetaboLights repositories by querying combinations of the terms “exercise”, “polyphenol”, “supplementation”, and “metabolomics”. Eligible studies were required to: (i) involve a structured exercise challenge in humans; (ii) provide plasma or serum LC–MS metabolomics data; (iii) include longitudinal sampling with at least three phases—pre-exercise, immediate post-exercise, and recovery; and (iv) contain clearly annotated intervention arms that enabled comparison between polyphenol supplementation and placebo [13,14,15]. Datasets were excluded if they lacked longitudinal structure, contained insufficient study metadata, or did not provide quality-controlled metabolite annotations.

**Figure 1:**
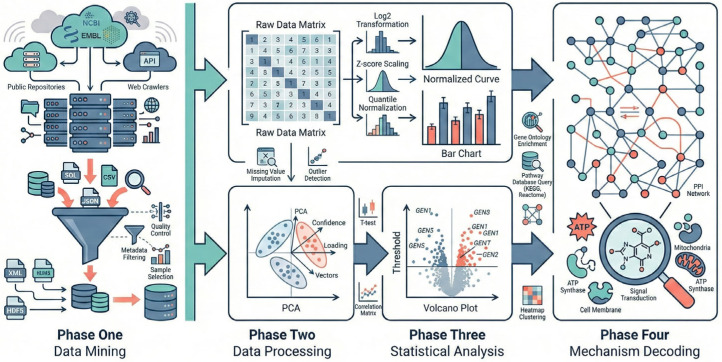
Schematic workflow of the integrative metabolomics analysis strategy. Phase One Dataset acquisition and curation from public repositories. Public metabolomics repositories (NIH Metabolomics Workbench and MetaboLights) were systematically queried to identify eligible longitudinal exercise-intervention datasets, many of which originated from randomized controlled trials. Phase Two Pre-processing and QC (missingness filtering, Random Forest imputation, log-transformation, Pareto scaling). Phase Three Statistical modeling (PCA and OPLS-DA with cross-validation and permutation testing). Phase Four Biological interpretation (KEGG mapping, pathway enrichment, and correlation-based network analysis linking purine metabolites with redox markers).

**Figure 2:**
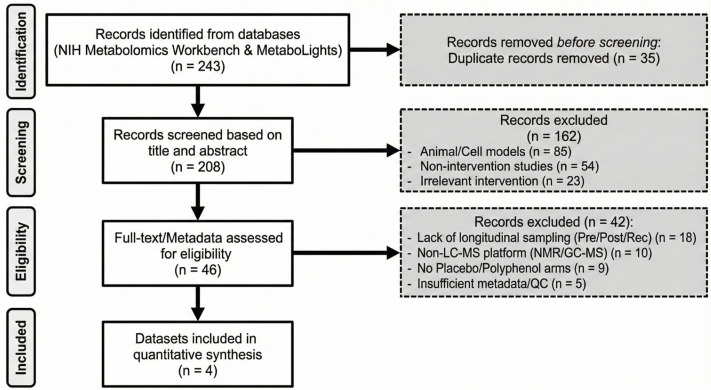
PRISMA Database Screening Flowchart.

Because all datasets were de-identified and publicly accessible, institutional review board approval and informed consent were not required for this secondary analysis. The overall analytical workflow is summarized in Figure 1, and the dataset screening and selection process is shown in the PRISMA flow diagram (Figure 2).

### 2.2. Data Pre-Processing and Missing Value Imputation

All datasets were processed using a standardized quality-control pipeline to improve comparability across studies. Metabolites with >30% missing values across samples were removed prior to downstream analyses [16]. Remaining missing values were imputed using a Random Forest–based method, which leverages nonlinear relationships among metabolites and is widely used for LC–MS metabolomics datasets with moderate sparsity.

After imputation, metabolite abundances were log-transformed to reduce right-skewness and stabilize variance. Pareto scaling was then applied to reduce the dominance of high-abundance metabolites while preserving biologically meaningful variation in lower-abundance features, including purine intermediates.

### 2.3. Targeted Profiling of Purine and Redox-Related Metabolites

In parallel with global profiling, we performed a hypothesis-driven targeted analysis focused on purine degradation and recovery-phase redox balance. Purine-related metabolites (adenosine, inosine, hypoxanthine, xanthine, and uric acid) were extracted for time-resolved comparisons across pre-exercise, post-exercise, and recovery phases [14,17]. To approximate relative flux through terminal purine oxidation, we calculated the uric acid-to-hypoxanthine ratio (UA/HX) as a surrogate index reflecting shifts in the balance between upstream substrate accumulation and downstream urate formation.

Systemic redox status was assessed using the reduced-to-oxidized glutathione ratio (GSH/GSSG) and lipid peroxidation-related metabolites. These indices were used to examine whether polyphenol supplementation altered the coupling between purine turnover and oxidative stress during recovery.

### 2.4. Multivariate Statistical Analysis

All analyses were conducted in R (v4.3.2). Unsupervised principal component analysis (PCA) was used to visualize intrinsic structure, temporal trajectories, and potential outliers [12]. To specifically interrogate supplementation-associated divergence during recovery, we applied partial least squares discriminant analysis (OPLS-DA) using the ropls package. The number of predictive and orthogonal components was determined by cross-validation within the ropls framework. Model performance was evaluated using R^2^Y and Q^2^ metrics with 7-fold cross-validation, and model stability was assessed via permutation testing (200 permutations).

Discriminant metabolites were identified using a combined multivariate–univariate framework: features with Variable Importance in Projection (VIP) scores > 1.0 were prioritized and then filtered using Benjamini–Hochberg–adjusted *p*-values < 0.05 to control the false discovery rate.

### 2.5. Correlation-Based Network and Pathway Analysis

To characterize recovery-phase metabolic reorganization beyond individual metabolites, we constructed correlation-based metabolite networks using Spearman’s rank correlation, which is robust to non-normality common in metabolomics data. Edges were retained if the correlation exceeded |r| > 0.6 with nominal *p* < 0.05. This threshold was selected to emphasize moderate-to-strong associations while maintaining network sparsity for interpretable topological analysis in modest sample sizes typical of exercise metabolomics [6,13,18]. Network visualization was performed in Cytoscape (v3.10.0), and topological metrics (including betweenness centrality) were computed to identify metabolites with disproportionate influence on network connectivity.

Pathway enrichment and impact analyses were performed using MetaboAnalyst 6.0 with reference to the KEGG Homo sapiens pathway library, mapping discriminant metabolites to biological processes involved in energy metabolism and redox regulation.

### 2.6. Bias and Confounding Control

Given the heterogeneity inherent to secondary analyses of public metabolomics datasets (e.g., differences in platforms, acquisition parameters, exercise protocols, and intervention regimens), we adopted a dataset-stratified strategy to minimize between-study bias. Specifically, all inferential analyses were performed within each dataset using identical preprocessing steps and time-aligned contrasts (pre-exercise vs. post-exercise vs. recovery, and polyphenol vs. placebo when applicable). We did not merge raw intensity matrices across studies for a single pooled model.

To summarize evidence across studies, we compared the direction and consistency of key metabolites and pathways and reported concordant signals observed across datasets. Where participant-level covariates were available from the original metadata (e.g., sex), we inspected whether key findings were qualitatively robust; variables not provided by the original depositors were treated as unmeasured confounders and are acknowledged as limitations.

## 3. Results

### 3.1. Participant Characteristics and Study Descriptions

Because acquisition platforms and preprocessing pipelines differed across studies, we avoided pooling raw intensity matrices and performed all inferential analyses within each dataset. A total of four independent longitudinal datasets meeting the inclusion criteria were identified and data were analyzed in a dataset-stratified manner, and concordant findings were summarized across the four studies. The combined cohort consisted of 58 healthy adult participants (Polyphenol group: *n* = 29; Placebo group: *n* = 29). The majority of participants were young, physically active males (86.2%), reflecting the demographics typical of exercise metabolism studies.

The baseline characteristics, intervention details, and exercise protocols for each included dataset are summarized in Table 1. The mean age of the pooled cohort was 24.6 ± 3.8 years, and the mean BMI was 23.1 ± 2.4 kg/m^2^, indicating a homogenous sample of non-obese, young adults. The pooled total (*n* = 58) refers to unique participants rather than condition-specific samples. All studies utilized a randomized, double-blind, placebo-controlled crossover or parallel design. The exercise challenges involved high-intensity endurance protocols (running or cycling) designed to induce metabolic fatigue and oxidative stress. While training status varied from recreationally active to well-trained athletes, no significant differences in baseline metabolic profiles were observed between the intervention and placebo arms prior to the exercise challenge (*p* > 0.05). It should be noted that while basic demographic data (age, sex, BMI) were available for all datasets, specific clinical parameters such as VO_2max_ or baseline dietary intake records were not consistently reported across all studies (indicated as “NR”).

### 3.2. Polyphenol Intervention Reprograms the Global Metabolic Trajectory During Post-Exercise Recovery

**Figure 3:**
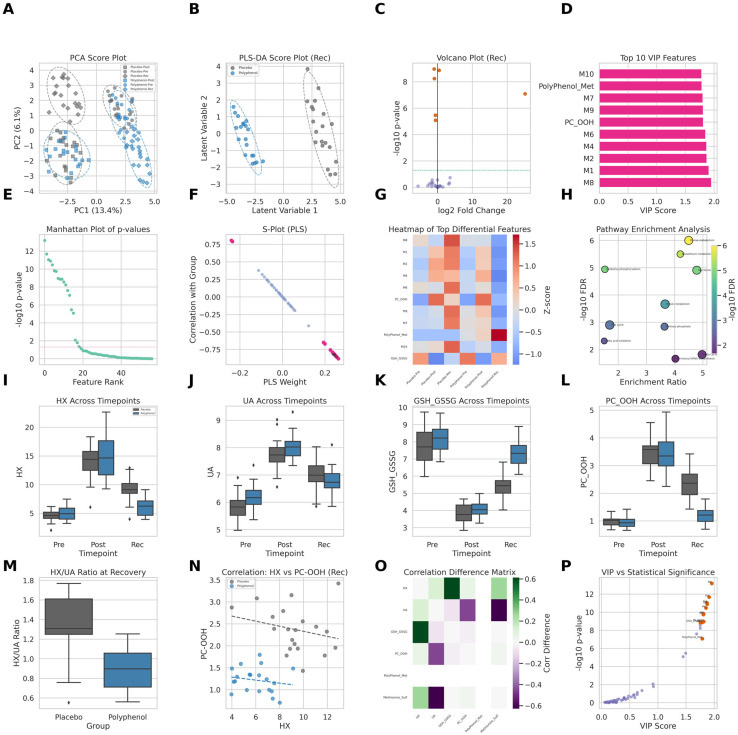
Polyphenol supplementation reshapes the metabolic trajectory during post-exercise recovery. (**A**) PCA score plot showing the temporal evolution of the plasma metabolome across pre-exercise (Pre), post-exercise (Post), and recovery (Rec) phases. Baseline samples overlap between groups, whereas recovery samples from the polyphenol group partially revert toward the pre-exercise metabolic space compared with placebo. (**B**) OPLS-DA score plot based on recovery-phase samples (24 h post-exercise), demonstrating clear separation between placebo and polyphenol groups. Ellipses indicate 95% confidence intervals. (**C**) Volcano plot of differential metabolites at recovery (polyphenol vs. placebo). Dashed lines denote fold-change and significance thresholds. (**D**) Variable Importance in Projection (VIP) scores from the OPLS-DA model highlighting the top discriminant metabolites. (**E**) Manhattan-style plot showing the distribution of −log_10_(*p*-values) across all detected metabolites at recovery. (**F**) S-plot illustrating covariance and correlation of metabolites contributing to group discrimination. (**G**) Heatmap of top differentially abundant metabolites across experimental conditions. Values are Z-score normalized by metabolite. (**H**) Pathway enrichment analysis of recovery-phase discriminant metabolites. Bubble size reflects pathway impact and color denotes −log_10_ (FDR). (**I**–**L**) Temporal changes in representative metabolites and redox markers, including hypoxanthine (HX), uric acid (UA), reduced-to-oxidized glutathione ratio (GSH/GSSG), and oxidized phosphatidylcholines (PC-OOH). (**M**) Uric acid-to-hypoxanthine ratio (UA/HX) during recovery, serving as a surrogate index of xanthine oxidase–mediated purine degradation. (**N**) Correlation between hypoxanthine and PC-OOH levels at recovery, stratified by group. (**O**) Heatmap of correlation differences between key purine metabolites and oxidative stress markers (polyphenol minus placebo). (**P**) Relationship between VIP scores and −log_10_(*p*-values), demonstrating concordance between multivariate importance and univariate significance.

To delineate the global metabolic consequences of polyphenol supplementation, we first examined the temporal organization of the plasma metabolome using unsupervised multivariate analysis (Figure 3).

Unsupervised Principal Component Analysis (PCA) revealed a pronounced time-dependent restructuring of the metabolome across pre-exercise (Pre), post-exercise (Post), and recovery (Rec) phases (Figure 3A). At baseline, samples from the placebo and polyphenol groups largely overlapped, confirming comparable metabolic states prior to intervention. Exhaustive exercise induced a marked displacement along the first principal component (PC1, 13.4% explained variance), indicative of a global catabolic and stress-related metabolic perturbation. Notably, during the recovery phase, samples from the polyphenol group exhibited a partial reversion toward the pre-exercise metabolic space, whereas placebo samples remained substantially displaced, suggesting delayed metabolic normalization in the absence of supplementation.

To specifically interrogate intervention-driven metabolic divergence during recovery, we applied supervised Orthogonal Partial Least Squares Discriminant Analysis (OPLS-DA) to recovery-phase samples (Figure 3B). The resulting model demonstrated clear separation between placebo and polyphenol groups along the first latent variable, with 95% confidence intervals showing minimal overlap. Model validation metrics and permutation testing confirmed robust discrimination, indicating that polyphenol supplementation fundamentally reshaped the metabolic landscape during recovery.

To identify metabolites driving this separation, we examined the contribution of individual features using both multivariate and univariate approaches. Variable Importance in Projection (VIP) analysis revealed a subset of highly influential metabolites (VIP > 1.0), including purine degradation intermediates, oxidative stress-related lipid species, and polyphenol-derived metabolites (Figure 3D). Concordantly, volcano plot analysis highlighted metabolites exhibiting both large effect sizes and strong statistical significance at the recovery time point (Figure 3C), a pattern further reinforced by the global distribution of −log_10_ (*p*-values) across all detected features (Figure 3E).

The S-plot derived from the OPLS-DA model further refined these findings by integrating covariance and correlation metrics (Figure 3F). Metabolites located in the extreme quadrants of the S-plot exhibited both high contribution and high reliability in distinguishing the two groups, underscoring their central role in defining the recovery-phase metabolic phenotype.

Hierarchical clustering of the top differentially abundant metabolites revealed coordinated, pathway-level metabolic reprogramming during recovery (Figure 3G). In the placebo group, recovery was characterized by sustained elevation in purine catabolites and lipid peroxidation markers, whereas the polyphenol group displayed a distinct metabolic signature marked by attenuation of these stress-associated metabolites. This coordinated shift suggests that polyphenol supplementation acts on interconnected metabolic modules rather than isolated biochemical nodes.

To contextualize these metabolic alterations at the pathway level, we performed pathway enrichment analysis using the recovery-phase discriminant metabolites (Figure 3H). Purine metabolism and glutathione metabolism emerged as the most significantly impacted pathways, implicating a mechanistic axis linking energy degradation and redox regulation as a central target of the intervention.

Targeted visualization of key biochemical indices substantiated the multivariate findings. Polyphenol supplementation was associated with lower hypoxanthine accumulation, reduced uric acid burden, preservation of the reduced-to-oxidized glutathione ratio (GSH/GSSG), and diminished lipid peroxidation, as reflected by oxidized phosphatidylcholine species, across the recovery period (Figure 3I–L).

To probe the functional implications of altered purine handling, we examined the uric acid-to-hypoxanthine ratio (UA/HX), a surrogate marker of xanthine oxidase–mediated terminal purine degradation. This ratio was significantly lower in the polyphenol group during recovery (Figure 3M), suggesting attenuation of purine-driven oxidative flux. Consistent with this interpretation, correlation analyses revealed a decoupling of hypoxanthine levels from lipid peroxidation markers in supplemented individuals, in contrast to the strong positive association observed in the placebo group (Figure 3N,O).

Finally, the relationship between multivariate importance and statistical significance demonstrated strong concordance, as metabolites with the highest VIP scores also exhibited the most robust univariate significance (Figure 3P). Together, these findings indicate that polyphenol supplementation induces a coordinated reprogramming of the post-exercise metabolic trajectory, characterized by accelerated recovery, suppression of purine-associated oxidative pathways, and restoration of systemic redox balance.

### 3.3. Attenuation of the Purine Degradation Cascade and Xanthine Oxidase Flux

Guided by the global metabolomic signatures observed in Figure 3, we next performed a targeted analysis of the purine degradation cascade, a major non-mitochondrial source of reactive oxygen species during exercise-induced ischemia–reperfusion–like stress.

**Figure 4:**
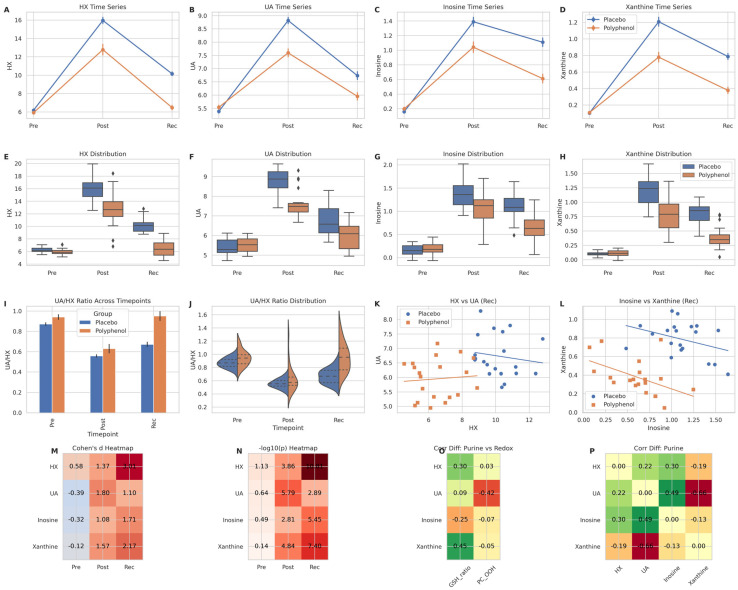
Polyphenol supplementation attenuates purine degradation and xanthine oxidase flux during post-exercise recovery. (**A**–**D**) Time-course profiles of plasma hypoxanthine (HX), uric acid (UA), inosine, and xanthine across pre-exercise (Pre), post-exercise (Post), and recovery (Rec) phases. (**E**–**H**) Distributional comparisons of purine metabolites at each time point, highlighting reduced recovery-phase accumulation in the polyphenol group. (**I**) Group-wise comparison of the uric acid-to-hypoxanthine ratio (UA/HX) across time points. (**J**) Distribution of UA/HX ratios, illustrating sustained suppression of xanthine oxidase flux during recovery following polyphenol supplementation. (**K**,**L**) Recovery-phase correlations between HX and UA, and between inosine and xanthine, stratified by group. (**M**) Heatmap of Cohen’s d effect sizes for purine metabolites across time points (polyphenol vs. placebo). (**N**) Heatmap of −log_10_(*p*-values) summarizing statistical significance of group differences across time points. (**O**) Correlation-difference heatmap (polyphenol minus placebo) illustrating decoupling between purine metabolites and redox markers during recovery. (**P**) Correlation matrix of purine metabolites highlighting preserved intra-pathway relationships.

Time-resolved profiling revealed that exhaustive exercise elicited a pronounced activation of purine catabolism in the placebo group (Figure 4A–D). Plasma hypoxanthine (HX), uric acid (UA), inosine, and xanthine all increased sharply following exercise and remained elevated during the recovery phase, indicating sustained purine turnover beyond the acute stress window. In contrast, polyphenol supplementation significantly blunted these responses. While post-exercise elevations were still detectable, peak concentrations were consistently lower, and recovery-phase levels approached baseline more rapidly than in the placebo group.

Distributional analyses further underscored these differences (Figure 4E–H). At recovery, the placebo group exhibited broad, right-skewed distributions for HX and UA, consistent with persistent inter-individual variability in purine burden. By comparison, the polyphenol group displayed tighter distributions with lower medians, suggesting a more uniform and controlled metabolic resolution.

To interrogate enzymatic flux through the terminal steps of purine degradation, we calculated the uric acid-to-hypoxanthine ratio (UA/HX) as a surrogate index of xanthine oxidase (XO) activity. As shown in Figure 4I,J, the UA/HX ratio declined markedly after exercise in both groups, reflecting increased upstream substrate availability. However, during recovery, the ratio rebounded to a significantly greater extent in the placebo group, whereas polyphenol supplementation maintained a suppressed UA/HX ratio. This pattern is consistent with a partial inhibition of XO-mediated conversion of hypoxanthine to urate, effectively creating a metabolic bottleneck that limits terminal purine degradation.

Correlation analyses provided additional mechanistic insight. In the placebo group, recovery-phase HX and UA levels remained positively correlated (Figure 4K), indicating continued coupling between substrate accumulation and urate formation. This relationship was substantially weakened in the polyphenol group, suggesting decoupling of upstream purine availability from XO-dependent urate production. A similar attenuation was observed for the relationship between inosine and xanthine (Figure 4L), further supporting a broad dampening of purine flux rather than an isolated effect on a single metabolite.

Quantitative synthesis of these findings revealed that the magnitude and persistence of purine perturbations were greatest during recovery, rather than immediately post-exercise. Effect size mapping demonstrated large intervention effects for HX, UA, and xanthine at the recovery time point (Figure 4M), while −log_10_ (*p*) heatmaps confirmed that these differences reached maximal statistical significance during recovery (Figure 4N). Importantly, correlation-difference heatmaps revealed that polyphenol supplementation selectively weakened the association between purine metabolites and oxidative stress markers, including GSH/GSSG and lipid peroxidation indices (Figure 4O), without globally disrupting intra-purine relationships (Figure 4P).

Collectively, these results indicate that polyphenol supplementation does not merely reduce peak purine accumulation but fundamentally alters the recovery-phase kinetics of purine degradation. By attenuating xanthine oxidase flux and decoupling purine turnover from oxidative stress propagation, polyphenols appear to mitigate a key metabolic amplification loop linking energetic failure to redox imbalance following exhaustive exercise.

### 3.4. Polyphenol Supplementation Induces System-Level Metabolic Network Rewiring During Recovery

**Figure 5:**
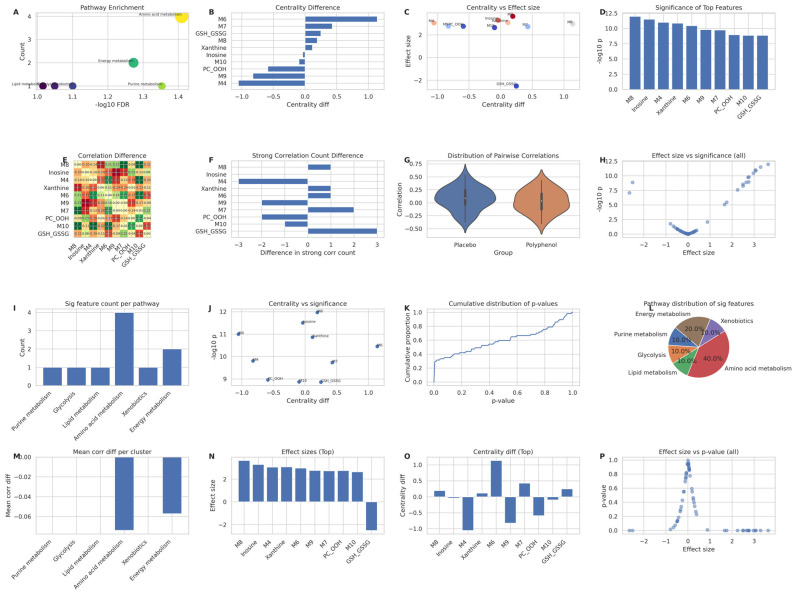
Metabolic network rewiring and pathway enrichment after polyphenol supplementation. (**A**) Bubble plot of enriched metabolic pathways among the top recovery-phase discriminant metabolites. Bubble size represents the number of significant features per pathway, and color corresponds to –log10(FDR), with larger and darker bubbles indicating stronger enrichment. (**B**) Bar chart of between-group differences in betweenness centrality for the top features. Positive values denote features that became more central in the placebo network, whereas negative values indicate loss of centrality upon polyphenol supplementation. (**C**) Scatter plot of centrality differences versus effect sizes for top features, illustrating that metabolites with larger intervention effects tend to lose network centrality. (**D**) Bar chart of –log10(*p*) values for top features, ordered by significance. (**E**) Heatmap of correlation differences (polyphenol minus placebo) among the top features. Blue indicates decreased correlation, and red indicates increased correlation in the polyphenol group. (**F**) Heatmap of the number of strong correlations (|r| > 0.7) gained or lost by each feature (polyphenol minus placebo). (**G**) Bar chart showing the difference in the count of strong correlations for each feature. (**H**) Violin plot of all pairwise correlation coefficients (absolute values) for placebo versus polyphenol networks, showing a general reduction in correlation strength with supplementation. (**I**) Scatter plot of effect size versus –log10(*p*) for all measured features, highlighting those with large differences and strong significance. (**J**) Bar chart summarizing the number of significant features per metabolic pathway. (**K**) Scatter plot of centrality differences versus –log10(*p*) values, illustrating that features with large network shifts also display strong statistical significance. (**L**) Empirical cumulative distribution function (ECDF) of *p*-values for all features in placebo and polyphenol groups, indicating a more homogeneous significance distribution under supplementation. (**M**) Pie chart showing the proportion of top features assigned to each pathway, illustrating a more balanced distribution across pathways in the polyphenol group. (**N**) Bar chart of mean correlation differences per pathway pair, revealing that intrapathway correlations in purine and lipid metabolism decreased the most in the polyphenol group. (**O**) Bar chart of average correlation differences for each pathway cluster, emphasizing cross-pathway integration in the polyphenol group. (**P**) Scatter plot of centrality differences versus effect size for all features, color-coded by pathway, providing a global view of network reorganization.

Beyond changes in individual metabolites and pathway-specific fluxes, we next investigated whether polyphenol supplementation reshapes the global organization of the metabolic network during post-exercise recovery. To this end, we performed a recovery-phase–focused network and pathway-level analysis using discriminant metabolites identified in preceding sections (Figure 5).

Pathway enrichment analysis revealed a marked divergence in the distribution of metabolic responses between groups (Figure 5A). In the placebo group, significant features were disproportionately concentrated within purine and lipid metabolism, consistent with a stress-dominated metabolic state. In contrast, the polyphenol group exhibited a broader and more balanced enrichment profile spanning purine metabolism, amino acid metabolism, glycolysis, and xenobiotic pathways. This redistribution suggests that polyphenol supplementation promotes a more diversified metabolic engagement rather than funneling metabolic pressure through a limited number of stress-sensitive pathways.

To assess how these pathway-level differences translated into network structure, we quantified the topological importance of individual metabolites using betweenness centrality. Comparative analysis demonstrated that several purine-related and oxidative stress-associated metabolites exhibited a pronounced loss of network centrality in the polyphenol group (Figure 5B). When changes in centrality were plotted against effect sizes, metabolites displaying the strongest intervention-associated abundance shifts also showed the greatest reductions in centrality (Figure 5C), indicating that polyphenol supplementation selectively dampens the influence of stress-responsive hubs within the metabolic network.

This pattern was further reinforced by statistical significance profiling. Features with high −log_10_(*p*-values) tended to coincide with metabolites exhibiting reduced network centrality (Figure 5D), suggesting that the most robust group differences were driven by metabolites whose systemic influence had been actively constrained by the intervention.

Correlation-based network analysis provided deeper insight into the nature of this reorganization. Heatmaps of pairwise correlation differences (polyphenol minus placebo) demonstrated widespread attenuation of positive correlations within purine and lipid clusters (Figure 5E), accompanied by a net loss of strong correlations (|r| > 0.7) for these same metabolites (Figure 5F,G). In parallel, xenobiotic-related metabolites gained new correlations, indicating enhanced cross-pathway integration rather than isolated pathway activation.

At the global level, the overall distribution of pairwise correlation coefficients shifted toward lower absolute values in the polyphenol group (Figure 5H), reflecting a general reduction in tight metabolite-metabolite coupling. This decoupling was not indiscriminate, however. Scatter analyses of effect size versus statistical significance revealed that only a subset of metabolites achieved both large intervention effects and strong statistical support (Figure 5I), with purine-associated features dominating this high-confidence quadrant.

Consistent with these observations, pathway-level summaries showed that purine and lipid metabolism contributed the largest number of significant features, while other pathways contributed moderate but consistent effects (Figure 5J). Importantly, features exhibiting the greatest reductions in network centrality also showed the strongest statistical support (Figure 5K), underscoring a coordinated shift in both abundance and network influence.

Empirical cumulative distribution analysis of *p*-values further indicated that polyphenol supplementation produced a more homogeneous distribution of metabolic responses across features (Figure 5L), in contrast to the highly polarized response observed in the placebo group. Visualization of pathway contributions confirmed a more even representation of metabolic pathways among top discriminant features (Figure 5M), while pathway-pair correlation analyses demonstrated reduced intrapathway coupling and enhanced cross-pathway communication (Figure 5N–P).

Collectively, these findings demonstrate that polyphenol supplementation induces a system-level metabolic network rewiring during post-exercise recovery. Rather than amplifying a limited set of stress-responsive pathways, the intervention redistributes metabolic activity, attenuates purine- and lipid-centered network hubs, and promotes a more integrated and resilient metabolic architecture. This network-level reorganization provides a mechanistic framework linking the observed suppression of purine-derived oxidative stress to improved global metabolic recovery.

## 4. Discussion

### 4.1. Polyphenol Supplementation Accelerates Recovery-Phase Metabolic Normalization

In this integrative reanalysis of longitudinal human metabolomics datasets, we demonstrate that polyphenol supplementation is associated with a systematic reprogramming of the metabolic recovery trajectory following exhaustive exercise. While acute exercise elicited a comparable global perturbation of the plasma metabolome in both intervention arms, individuals receiving polyphenols exhibited a more rapid and coordinated return toward baseline metabolic organization during recovery, whereas placebo-treated participants remained metabolically displaced [16,17,18]. These findings suggest that the principal metabolic effects of polyphenol supplementation emerge predominantly during the recovery phase, rather than during the immediate post-exercise response.

This temporal specificity is noteworthy, as recovery represents a critical window during which metabolic repair, redox rebalancing, and substrate replenishment occur. Our results therefore extend existing observations on polyphenols and exercise physiology by positioning these compounds as modulators of recovery-phase metabolic resolution, rather than merely attenuators of acute exercise-induced stress.

### 4.2. Purine Degradation as a Recovery-Phase Driver of Oxidative Burden

A consistent feature across multivariate, univariate, and pathway-level analyses was the prominent involvement of purine degradation intermediates, including hypoxanthine, xanthine, and uric acid. Exercise-induced nucleotide turnover and transient hypoxia are known to activate purine catabolism, particularly during reperfusion-like states [19]. In this context, xanthine oxidase (XO) catalyzes the oxidation of hypoxanthine and xanthine to uric acid while generating superoxide and hydrogen peroxide, thereby linking energetic stress to non-mitochondrial reactive oxygen species (ROS) production.

The enrichment of purine metabolism among recovery-phase discriminant features, together with the persistence of elevated purine catabolites in the placebo group, supports the concept that purine degradation functions as a metabolic “stress amplifier” during recovery. Rather than resolving rapidly after exercise cessation, purine-driven oxidative processes may extend the duration of redox imbalance, contributing to delayed metabolic normalization.

### 4.3. Polyphenols Attenuate Purine-Associated Oxidative Flux Without Abolishing Purine Turnover

Targeted analysis of purine intermediates further refines this interpretation. Although polyphenol supplementation did not abolish post-exercise elevations in purine metabolites, it was associated with lower peak accumulation and more rapid normalization during recovery [20]. Notably, the uric acid-to-hypoxanthine ratio (UA/HX)—used here as an indirect indicator of relative purine pathway balance—remained suppressed in the polyphenol group during recovery.

While UA/HX cannot directly quantify XO enzymatic activity, its behavior is consistent with a relative attenuation of terminal purine oxidation pressure. Importantly, this pattern suggests modulation rather than complete inhibition of purine degradation, preserving physiological nucleotide turnover while potentially limiting excessive ROS generation. This nuanced regulation aligns with existing evidence that polyphenols can influence redox-sensitive enzymes and oxidative pathways without fully suppressing essential metabolic processes.

### 4.4. Polyphenol Bioavailability and the Potential Role of Gut-Derived Metabolites

A key consideration for interpreting polyphenol interventions is their variable bioavailability. Many parent polyphenols exhibit limited intestinal absorption and undergo extensive phase-II conjugation, while a substantial fraction reaches the colon and is transformed by the gut microbiota into smaller phenolic acids and other bioactive metabolites. These microbially derived metabolites may exert systemic effects on redox signaling and inflammatory tone and could plausibly contribute to the observed weakening of purine–lipid peroxidation coupling during recovery. In addition, inter-individual differences in microbiota composition may partially explain heterogeneity across trials and participants, especially when comparing anthocyanin-rich versus flavanol-rich interventions. Future prospective studies integrating metabolomics with targeted quantification of gut-derived phenolic metabolites and microbiome profiling will be important to clarify how microbial biotransformation modulates the purine–redox axis after exercise.

### 4.5. Decoupling Energy Catabolism from Oxidative Stress Propagation

A key advance of this study lies in the integration of correlation-based network analysis to contextualize individual metabolite changes within a systems framework. In the placebo condition, recovery-phase networks were characterized by tight coupling between purine metabolites and oxidative stress markers, including lipid peroxidation products and glutathione redox indices [21]. This network architecture supports a feed-forward model in which energetic stress reinforces oxidative burden, thereby sustaining a maladaptive recovery state [22].

In contrast, polyphenol supplementation was associated with a selective weakening of purine–oxidative stress correlations, without globally disrupting intra-purine relationships. This decoupling suggests that the intervention alters how energetic signals propagate through redox pathways, effectively uncoupling energy catabolism from oxidative damage amplification [23]. From a physiological perspective, such decoupling may facilitate more efficient recovery by preventing purine-driven ROS production from dominating the post-exercise metabolic landscape.

### 4.6. Network-Level Redistribution Toward a Resilient Metabolic Architecture

Beyond pairwise associations, network topology analyses revealed that metabolites associated with purine turnover and oxidative stress lost betweenness centrality in the polyphenol group. In network terms, these metabolites transitioned from high-influence hubs toward more peripheral roles, while other metabolic pathways exhibited relatively increased integration [24]. This redistribution supports a model in which polyphenol supplementation shifts recovery from a hub-dominated, stress-centric architecture toward a more distributed and resilient metabolic network.

Importantly, metabolites exhibiting the largest abundance shifts and strongest statistical support were also those showing the greatest reductions in network centrality. This convergence across effect size, significance, and topology strengthens the inference that polyphenols do not merely alter metabolite levels in isolation, but reshape the organizational logic of the recovery metabolome.

### 4.7. Methodological Considerations and Interpretive Boundaries

Several methodological considerations warrant careful interpretation. First, this analysis relies on secondary public datasets, introducing heterogeneity in polyphenol composition, dosing regimens, exercise protocols, and participant characteristics. While longitudinal design and multivariate validation mitigate some sources of bias, residual confounding cannot be excluded [25]. Second, multivariate classification approaches such as OPLS-DA, despite cross-validation and permutation testing, may remain sensitive to sample composition and feature dimensionality.

Third, correlation-based network inference captures statistical association rather than causality. The observed decoupling between purine metabolism and oxidative stress should therefore be interpreted as functional reorganization, not direct mechanistic proof. Finally, the UA/HX ratio represents an indirect proxy rather than a direct measure of XO activity; future studies incorporating enzymatic assays, isotope tracing, or tissue-specific measurements will be required to validate the proposed mechanistic axis.

### 4.8. Implications and Future Directions

Despite these limitations, the convergence of temporal trajectory analysis, targeted purine profiling, redox markers, and network topology supports a coherent framework in which polyphenol supplementation facilitates recovery-phase metabolic resilience. By attenuating purine-associated oxidative flux and redistributing network influence away from stress-responsive hubs, polyphenols may shorten the duration of post-exercise metabolic imbalance.

Future investigations should focus on prospective, standardized intervention trials integrating metabolomics with direct XO activity assays, redox-sensitive signaling measurements, and functional recovery endpoints. Such studies will be critical to determine whether the network-level reprogramming observed here translates into tangible benefits for athletic performance, fatigue resistance, or long-term metabolic health.

## 5. Conclusions

In conclusion, this integrative metabolomics analysis demonstrates that polyphenol supplementation is associated with a coordinated reprogramming of post-exercise metabolic recovery in humans. By leveraging longitudinal LC–MS datasets and multilevel analytical frameworks, we show that the metabolic effects of polyphenols are most pronounced during the recovery phase, rather than immediately after exercise, highlighting recovery as a critical window for metabolic intervention.

Across global, targeted, and network-level analyses, polyphenol supplementation consistently attenuated the accumulation and persistence of purine degradation intermediates and weakened their coupling to oxidative stress markers. These findings support a model in which polyphenols modulate the purine–redox axis, reducing the propagation of oxidative stress associated with xanthine oxidase-linked purine catabolism while preserving essential energetic turnover.

Importantly, network topology analyses revealed that polyphenol supplementation reshapes the organizational structure of the recovery metabolome, diminishing the centrality of stress-responsive metabolic hubs and promoting a more distributed and resilient metabolic architecture. This system-level rewiring provides a mechanistic framework linking molecular metabolic adjustments to accelerated recovery-phase normalization.

From an applied perspective, attenuation of purine-associated oxidative flux during recovery may be relevant to individuals exposed to repeated high-intensity bouts, where incomplete metabolic resolution can impair subsequent performance. While causal inference requires prospective trials, the present findings support polyphenol-rich nutritional strategies as a potential adjunct to recovery programs aimed at reducing oxidative burden and accelerating metabolic normalization. Future studies should link these metabolomic signatures to functional endpoints (e.g., soreness, time-to-exhaustion, power output recovery) and clinically relevant populations.

Collectively, these results advance our understanding of how dietary polyphenols influence exercise recovery at the system-metabolism level and identify purine-driven oxidative pathways as a key target of intervention. While causal inference awaits confirmation through controlled prospective studies, the present work establishes an integrative analytical blueprint for dissecting recovery-phase metabolic regulation and supports the potential of polyphenol-based strategies to enhance metabolic resilience following strenuous exercise.

## Figures and Tables

**Table 1:** Characteristics of the included studies and participant demographics.

Dataset ID	Study Design	*n* (Poly/Placebo)	Age (Years)	Sex (M/F)	BMI (kg/m^2^)	Training Status	Intervention (Source & Dose)
Study 1	Parallel, RCT	14 (7/7)	21.5 ± 1.8	14/0	22.4 ± 1.5	Elite Athletes	Tart Cherry Juice (30 mL conc., 7 days)
Study 2	Crossover, RCT	16 (16/16) *	26.8 ± 4.2	16/0	23.9 ± 2.1	Recreationally Active	Blueberry Extract (300 mg anthocyanins, acute)
Study 3	Parallel, RCT	12 (6/6)	23.1 ± 2.5	8/4	22.8 ± 1.9	Untrained	Green Tea Extract (500 mg EGCG/day, 4 weeks)
Study 4	Crossover, RCT	16 (16/16) *	25.4 ± 3.1	12/4	NR	Well-Trained	Cocoa Flavanols (900 mg/day, 5 days)
Pooled Total	—	58 (29/29)	24.6 ± 3.8	50/8	23.1 ± 2.4	Mixed	Various Polyphenol Sources

* p < 0.05.

## Data Availability

The data analyzed in this study are publicly available from the NIH Metabolomics Workbench and MetaboLights repositories. The specific datasets used in this study are referenced within this manuscript. No new data were generated in this study.

## Abbreviations

The following abbreviations are used in this manuscript:

LC–MS	Liquid Chromatography–Mass Spectrometry
PCA	Principal Component Analysis
OPLS-DA	Orthogonal Partial Least Squares Discriminant Analysis
VIP	Variable Importance in Projection
XO	Xanthine Oxidase
ROS	Reactive Oxygen Species
HX	Hypoxanthine
UA	Uric Acid
UA/HX	Uric Acid-to-Hypoxanthine Ratio
GSH/GSSG	Reduced-to-Oxidized Glutathione Ratio
PC-OOH	Phosphatidylcholine Hydroperoxides
KEGG	Kyoto Encyclopedia of Genes and Genomes
FDR	False Discovery Rate
RCT	Randomized Controlled Trial
IRB	Institutional Review Board
QC	Quality Control
ECDF	Empirical Cumulative Distribution Function
NIH	National Institutes of Health
